# Mechanochemical prepared ibuprofen-*Polygonatum sibiricum* polysaccharide drug delivery system for enhanced bioactivity with reduced renal injury induced by NSAIDs

**DOI:** 10.1080/10717544.2022.2026533

**Published:** 2022-01-20

**Authors:** Wenhao Xu, Jinli Yang, Xiangyang Gu, Wenjing Su, Faxiang Pu, Zhangfu Xie, Kongliang Jin, Weike Su, Lichan Mao

**Affiliations:** a Collaborative Innovation Center of Yangtze River Delta Region Green Pharmaceuticals, Zhejiang University of Technology, Hangzhou, China; b Huadong Medicine Co., Ltd., Hangzhou, China; c Zhejiang Suichang Limin Pharmaceutical Co., Ltd., Lishui, China; d Hangzhou Hospital of Traditional Chinese Medicine, Hangzhou, China

**Keywords:** Ibuprofen, *Polygonatum sibiricum* polysaccharide, drug delivery, mechanochemistry, drug nephrotoxicity

## Abstract

Ibuprofen (IBU) was a widely used NSAID (a type of nonsteroidal anti-inflammatory drug) worldwide, and many drug deliveries had been reported to enhance bioavailability. However, higher bioavailability would increase the danger of renal injury caused by oxidative stress. This study prepared IBU-*Polygonatum sibiricum* polysaccharide (IBU-PSP) drug delivery system via mechanochemical method. Due to drug delivery and renal protection effect of *Polygonatum sibiricum* polysaccharide (PSP), the solubility of IBU-PSP was increased 8.22 times, and the bioavailability was increased 2.52 times compared with IBU, carrageenin-induced rat paw edema test also increased. Meanwhile, short-term and long-term renal injuries induced by IBU were notable decreases. In conclusion, IBU-PSP was a multifunctional drug delivery system with superior anti-inflammatory and renal protection effects. It will benefit from developing high-efficiency NADIs preparations with safer clinical applications while providing an efficient and energy-saving technology for polysaccharide drug delivery.

## 1. Introduction

Ibuprofen (IBU) is a type of nonsteroidal anti-inflammatory drug (NSAIDs) with the largest use level at present (Pham et al., 2019). However, as a biopharmaceutics classification system II (BSC II) drug, the equilibrium solubility of IBU in water is only 50 μg/mL, thereby leading to a lower bioavailability, longer onset time, and lower actual medication effect (Alvarez et al., 2011). Therefore, the main idea of the research on IBU preparations is the quick-acting medicament form for improving the solubility and bioavailability of IBU in an aqueous solution. At present, many IBU solubilization technologies and IBU preparations with high bioavailability have been reported (Stephenson et al., 2006; Baek et al., 2012). However, a large number of studies that focused on clinical medication studies have discovered that during the process of renal metabolism, NSAIDs, such as IBU, can inhibit the synthesis of cyclooxygenase (COX) in the kidney (Lianos et al., 1983; Lawrence et al., 2008; Schrör, 2011) and cause oxidative stress and inflammation in the kidney (Basivireddy et al., 2004; Fattori et al., 2017), which results in the decrease of renal blood volume, renal ischemia, and a reduction in the glomerular filtration rate (GFR) (Lawrence et al., 2008), thereby leading to drug-induced renal damage (Rao et al., 1994; Chamaa et al., 2000; Hörl, 2010). The existing new IBU with a modified structure (Katritzky et al., 2009; Ahmadi et al., 2017) and its preparations can significantly improve the bioavailability of IBU in the human body.

Moreover, the plasma concentration of IBU can be increased 2–10-fold compared to that of the API, and its half-life could be prolonged (Khvostov et al., 2017). However, a high concentration of IBU in the body will be metabolized by the kidney, thereby greatly increasing the probability of kidney damage caused by IBU. Thus, improving the bioavailability of IBU and reducing the nephrotoxicity caused by the increase of the plasma concentration simultaneously are critical in the research and development of IBU and other NSAIDs.

Given the problems mentioned above, many researchers have tried solving the problems in nephrotoxicity caused by NSAIDs by using compound preparations (İlbey et al., 2009; Ortiz et al., 2010; Gao et al., 2019; Motaharinia et al., 2019); it was demonstrated that the extracts of *Phyllanthus amarus Sch, umach. & Thonn* (Adeneye & Benebo, 2008), *Trifolium repens* leaf extract (Ahmad & Zeb, 2020), and *Salacia oblonga* (Palani et al., 2011), when combined with NSAIDs, can exert their unique pharmacological effects as well as protect the kidney. The examples mentioned above indicate that the adoption of compound pharmaceutical preparations and non-effective components with a kidney protective function will be a breakthrough in the research on IBU preparations with high efficiency and low nephrotoxicity.

*Polygonatum sibiricum* is a traditional Chinese medicine for renal protection and treating nephropathy (Bibi et al., 2016; Cai et al., 2019; Zhao et al., 2019; Han et al., 2020; Wang et al., 2020). It has been widely used as a functional food around all Asian countries. *Polygonatum sibiricum* polysaccharide (PSP) is the main activity ingredient in *Polygonatum sibiricum*. It contains four polysaccharides with different molecular weights, and the average molecular weight is 41 kDa (Zhu et al., 2015). Exonuclease hydrolysis experiments showed that PSP was a highly branched polysaccharide like fructofuranosan and arabinogalactan (AG) (Liu et al., 2018; Zhao et al., 2020). It will be easy for PSP to form an intermolecular complex with insoluble molecules and realize drug transportation.

Nevertheless, PSP is dampened easily and unstable in high temperatures; PSP drug delivery by traditional methods will face inhomogeneous and agglomerate problems. It would be highly affected drug release. Recently, mechanochemistry was successfully applied to prepare highly efficient drug delivery systems based on polysaccharides. Chistyachenko et al. (2015) prepared a drug delivery system of aspirin via mechanochemical solid-phase technology. By using highly branched natural polysaccharide as the carrier, the solubility of aspirin increased 1.2-fold, and its anticoagulant effect increased twofold. Xu et al. (2021) used a planetary ball mill to prepare solid dispersion of 5-aminosalicylic acid with chitosan and sodium alginate as carriers, which formed a hydrogel in aqueous solution and could realize localized release in the colon, thereby achieving the purpose of treating ulcerative colitis. Kong et al. (2017) used mechanical ball milling technology to prepare a variety of compounds with different natural polysaccharides as carriers to solve the problems of poor water solubility and low oral bioavailability of Statins. The results showed that the three systems improved the water solubility and bioavailability of drugs and significantly reduced blood lipid than the original drugs. Hence, applying mechanochemistry to drug delivery will provide a new approach for preparing PSP drug deliveries.

The above studies fully indicate that developing a safer and more effective IBU delivery system requires a highly efficient preparation method and a pharmaceutical excipient that can increase the bioavailability of IBU and reduce the nephrotoxicity of IBU. Cai et al. (2019) found that PSP could reduce inflammatory cytokines and promote glucose uptake by promoting the expression of Nrf2. Li et al. (2018) found that PSP can protect against acute kidney injury by inhibiting THE P38 MAPK/ATF2 pathway. While, in this study, PSP was used as the polymer carrier to prepare the IBU-PSP delivery system by mechanochemistry. With a kidney protective effect and good hydrophilicity of PSP, the bioavailability and anti-inflammatory effect of IBU can be increased, and kidney injury caused by IBU with high plasma concentration can be inhibited, and application of mechanochemistry will simultaneously supply an excellent solution for developing PSP based complex. It is significant for developing an efficient and low-toxicity drug delivery system with an easy and energy-saving process.

## 2. Materials and methods

### 2.1. Reagents and materials

Ibuprofen (purity 98%) was purchased from Aladdin Biotechnology Co., Ltd. (Xian, China). *Polygonatum sibiricum Red*. were collected in April 2020 in Chizhou, Anhui, China, and were identified by Dr. Yi Tao from Zhejiang University of Technology. (+)-Arabinogalactan (AG) from Larch Wood (purity 99%, CAS 9036-66-2) was purchased from Beijing Budweiser Technology Co., Ltd. (Beijing, China). Carrageenan (purity 98%, CAS 9000-07-1) was purchased from Aladdin Biotechnology Co., Ltd. (Xian, China). The XOD and ADA assay kits were produced by Nanjing Jiancheng Biotech (Nanjing, China). Malondialdehyde (MDA), superoxide dismutase (SOD), TUNEL kits, and uric acid (UA), creatinine (Cr), blood urea nitrogen (BUN), and adenosine deaminase (ADA) assay kits were from Beyotime Biotechnology (Haimen, China). All other chemicals used were of analytical grade.

### 2.2. Extraction and characterize of PSP

Polygonatum polysaccharide (PSP) was extracted from the root of *Polygonatum sibiricum* Red. by water, after ethanol precipitation and deproteinated by the Savag method, the total polysaccharide in the sample was in the sample 98.02%, gel permeation chromatography (GCP) measurement showed that the molecular weight of the main compound in PSP was 46.02 kDa.

### 2.3. Preparation of IBU-PSP and IBU-AG

As a green and efficient method, mechanochemical solid-phase preparation technology is widely used to prepare solid dispersions and high-performance materials (Zhang et al., 2021; Su et al., 2021). In this report, the solid dispersion of IBU-PSP was prepared by green mechanochemical approaches based on solvent-free ball milling techniques. Briefly, IBU, PSP, and adjuvants were finely mixed and added to a cylindrical ball mill tank (300 mL) coated with Teflon. A roller ball mill (ML 007, WIGGENS, Straubenhardt, Germany) was used, and the processing mode was as follows: grinding media − 50 steel balls (22 mm), rotation speed − 50–300 rpm, ball milling time – 0.5–4 h. Finally, the solubility of IBU in prepared samples was applied to screen the optimal formulation and process parameters.

Reported IBU solid dispersion with high bioavailability was prepared and used as a control. The preparation process of the repeated literature is as follows: IBU and AG were mixed in a mass ratio of 1:10, then add it to a 500 mL ball milling tank, and 1000 g, 22 mm stainless steel ball was added, then milled in a roller mill with 300 rpm for 4 h. The solubility of IBU in IBU-AG was up to 92.58 mg/L.

### 2.4. Content and solubility determinations

The method for determining the content of the drug in the SDs involved dissolving a certain amount of sample in 10 mL of methanol and then analyzing it by a high-performance liquid chromatography (HPLC) system (Shimadzu LC-20D, Kyoto, Japan) with an Ultimate XB-C18 column (4.6 × 150 mm, 5 μm) at 30 °C and an ultraviolet array detector. The eluent was CH_3_OH:0.1 mol/L H_3_PO_4_=65:35, with a 1.0 mL/min flow rate at a detection wavelength of 263 nm. All sample solutions were filtered through 0.45 μm filter paper, and the concentration of IBU was calculated as follows.

$$\tau_{\text{r}}=(-8.92\times 10^{-4}\gamma^{2}+0.094\gamma +4.07)\times 0.0131f_{\text{c}}$$

where PA is the peaking area of IBU; *V* is the dilution factor; *C*_IBU_ is the concentration of IBU solution.

Solubility tests were conducted on IBU, IBU-PSP, and IBU-AG. Excess samples were added to 10 mL of distilled water with stirring (200 rpm) for 12 h at 37 °C. Then, the sample solutions were filtered through 0.45 μm filter paper and analyzed by the above HPLC conditions.

### 2.5. Dissolution determination

A dissolution study was performed on the IBU SDs using a dissolution apparatus (Agilent Co. Ltd., Santa Clara, CA). Each sample (with an amount of IBU equivalent to 90 mg) was put into a dissolution vessel containing 900 mL of dissolution media, including pH 1.2 simulated gastric media and pH 6.8 phosphate buffer, and maintained at 37 ± 0.5 °C. Determination of the dissolution of all samples was performed in a dissolution tester at a paddle rotation speed of 100 rpm. At each predetermined time interval, 2 mL of sample solution was withdrawn, and an equal volume of fresh medium was added. The collected samples were filtered through a 0.45 μm microporous membrane and analyzed by HPLC.

### 2.6. X-ray diffraction (XRD) assay

The crystalline and amorphous behaviors of the samples were analyzed by XRD in an X-ray powder diffractometer (D2 PHASER, Bruker, Hamburg, Germany). The XRD patterns were recorded over a 2*θ* range of 5–80° with a step size of 0.026°.

### 2.7. Differential scanning calorimetry (DSC)

Thermal analysis of the prepared samples was carried out with a DSC-250 cell (Thermal 18 Analysis Co. Ltd., New Castle, DE) under an Ar atmosphere. Samples were placed in hermetically closed aluminum pans and heated from 50 °C to 300 °C.

### 2.8. Zeta potential and size measurements

The zeta potential and size of the samples were measured by dynamic light 111 scattering (DLS, Nano ZS90, Malvern 112 Instruments, Malvern, UK). The finely diluted samples in distilled water at 25 °C were analyzed for zeta potential 113 and size determination. The results were recorded as the average of sequential 20 measurements.

### 2.9. Scanning electron microscope (SEM) and transmission electron microscopy (TEM)

The solid-state morphology of IBU-PSP and other samples were studied by SEM (Gemini 500, Zeiss, Jena, Germany). Observations 127 were carried out with a voltage acceleration of 5 kV. Furthermore, the samples were also investigated with TEM (JEM 128 2100EX, JEOL, Tokyo, Japan) at an accelerating voltage of 100 kV. Sample images were obtained and analyzed by 129 JEM 2100EX.

### 2.10. ^1^H NMR spin–spin relaxation time *T*_2_ analysis

^1^H NMR spectra in solution were recorded by ‘Bruker’-NMR spectrometer AMD-500. The mixing-physically and mechanochemically prepared IBU-PSP and IBU-AG complexes were characterized in D_2_O solution (Aldrich, 99.8%, St. Louis, MO) with a concentration of 10 g/L. The spin–spin relaxation time *T*_2_ was measured using the standard Carr–Purcell–Meiboom–Gill (CPMG) sequence from Avance version of Bruker pulse sequence library: *P*_1_(90°) – (*τ* – *P*_2_(180°) – *τ*)*n* – registration, where *τ* = 0.5 ms is the fixed time delay, and *n* varied from 0 to 2000.

### 2.11. Pharmacokinetic study

Adult male Sprague-Dawley rats with a bodyweight of 250–275 g in the pharmacokinetic study were purchased from the Experimental Animal Center of Zhejiang Academy of Medical Sciences (production license number: SCXK-2019-0002). All experiments were approved by the Laboratory Animals Ethical Committee of the Zhejiang University of Technology and conformed to the National Institutes of Health Guide for care and use of laboratory animals (NIH publications no. 201907). SD rats were randomly divided into three groups, with six in each group: the IBU drug group, the IBU-AG drug delivery system, and the IBU-PSP drug delivery system group. IBU, IBU-AG, and IBU-PSP were administered orally at a dose of 25 mg/kg IBU/rat suspended in 0.5% sodium carboxymethylcellulose. At different time points at 15 min, 30, 45, 60, 90, and 120 min, about 500 µL of blood was taken from the rat orbital vein by a capillary (infiltrated with 1% heparin sodium) and placed in a centrifuge tube after washing with 1% heparin sodium. Then, blood was immediately centrifuged (Sigma Laboratory Centrifuge, Model 3K-30, Osterode am Harz, Germany) at 4 °C and 10,000 rpm for 10 min, and plasma was carefully separated and kept temporarily at −80 °C for further analysis. A total of 50 μL of rat plasma sample were accurately put into a 2-mL centrifuge tube, followed by adding 50 μL cinnamic acid (25 μg/L) as internal standard. After shaking for 15 min, 50 μL of methanol was added. Then, 100 μL of acetonitrile was added, and it was whirled for 1 min, followed by centrifugation for 10 min at 4 °C and 10,000 rpm. After standing, the supernatant was carefully sucked with a pipette for later use, and HPLC analyzed the treated plasma samples. The HPLC condition is shown in section 2.3.

### 2.12. *In vivo* anti-inflammatory efficacy

The carrageenan-induced foot swelling animal model was used to evaluate the anti-inflammatory activity of the preparation preliminarily. In brief, the specific method was as follows: 40 male SD rats, weighing 250–275 g, were randomly divided into model groups, including the IBU group, IBU-PSP drug delivery system group, and IBU-AG drug delivery system group, with 10 rats per group, and the experimental temperature was controlled at room temperature. At 1 h before edema was induced, SD rats were given normal saline, IBU (25 mg/kg), IBU-PSP delivery system (275 mg/kg), and IBU-AG delivery system (275 mg/kg) intragastrically, the solvent was 0.5% CMC-Na. Carrageenan was dissolved in 100 mL of normal saline, and a toe swelling model was established through injection into the left hind foot of SD rats with a 200 mg/paw dosage. Furthermore, the same amount of normal saline was injected into the right hind foot of SD rats as a control. A toe swelling detector was used to detect the increase in the degree of toe volume at 1, 2, and 4 h after drug stimulation to explore the change of inflammation degree through comparing with the model group.

### 2.13. *In vivo* renal protection efficacy

The acute kidney injury caused by IBU and IBU preparation was studied by increasing the dosage of IBU and drug samples. The specific experimental method was as follows: with 10 rats per group, 40 male SD rats weighing 250–275 g were randomly separated into four groups: normal, IBU, IBU-PSP administration system, and IBU-AG administration system. After one week of adaptive feeding, a series of acute kidney injury trials with high doses of IBU was given. The IBU group was given 180 mg/kg IBU intragastrically, the IBU-PSP drug delivery system group was administrated the IBU-PSP drug delivery system containing 180 mg/kg IBU through gavage, and the IBU-AG drug delivery system group was given IBU-AG drug delivery system containing 180 mg/kg IBU by gavage. The administration lasted for one week. During the drug administration test, rats had free access to food and water and were fed a common feed. At the end of the test, 1000 μL of blood was taken from the rats' orbital vein and placed in centrifuge tubes moistened with 1% heparin sodium, followed by immediate centrifugation at 4 °C and 10,000 rpm for 10 min. The serum was carefully removed with a pipette and put in a new centrifuge tube. An ELISA detection kit was used to measure the contents of serum creatinine (SCr), BUN in the serum.

The rats were deeply anesthetized with pentobarbital, and the kidney tissue was exposed by abdominal anatomy. After blood was replaced by perfusion through the heart with normal saline, the kidney on one side of the rats was removed and homogenized according to the ratio of tissue mass (g): PBS buffer volume (mL)=1:20. After centrifugation at 10,000×*g* at 4 °C for 10 min, the bottom sediment was discarded, and the supernatant protein concentration was determined. Then, levels of SCr, BUN, SOD, and MDA in the supernatant of tissue homogenate were determined by ELISA kits, and partial samples were collected for Western blot analysis.

At last, hematoxylin (HE) was used to stain the kidney tissue to study the changes in kidney microstructure. The whole kidney on the other side was taken out, and the kidney tissue taken after perfusion with normal saline and 4% paraformaldehyde was fixed in 10% neutral formalin solution. The kidney tissue fixed in 4% paraformaldehyde at 4 °C for 24 h was dehydrated and embedded in paraffin, and a freezing microtome cut 4 μm-thick sections. The paraffin sections were placed in an oven and baked at 65 °C for 50 min, then soaked in xylene (I) and (II) for 15 min, respectively. Hydration was carried out with different concentrations of ethanol (100, 90, 80, and 70%) for 2 min, respectively, followed by distilled water for 2 min. After rinsing the smear in PBS for 5 min, HE was employed to stain the nucleus for 10 min, followed by hydrochloric alcohol differentiation for 1 min, washing with distilled water for 1 min, eosin staining for 1 min, dehydration with 70, 85, 95% ethanol for 1 min, dehydration with 100% ethanol for 2 min/two times, as well as xylene transparency for 5 min/two times. Then, sections were observed under a microscope.

### 2.14. Long-term renal protection efficacy *in vivo*

The GFR is the most intuitive index for evaluating renal function. This study adopted a novel method to evaluate GFR, measuring the excretion kinetics of a single intravenous bolus of FITC-sinistrin (MediBeacon, Mannheim, Germany) percutaneously. Since FITC-sinistrin is a special fluorescent labeling substance that is only completely filtered by the glomeruli freely but not secreted or reabsorbed by renal tubules, this method can conduct accurate real-time detection on the GFR (Cai et al., 2019). The specific test scheme is as follows.

Male SD rats were placed in a specific-pathogen-free (SPF) laboratory animal room (with a temperature of 20–25 °C, and relative humidity of 50–60%) and fed a standard diet and water after 12 h of light/dark circulation. A total of 24 male SD rats were randomly divided into four groups, including a blank control group, IBU group, IBU-PSP drug delivery system group, and IBU-AG drug delivery system group, with six rats per group. IBU (50 mg/kg), IBU-PSP delivery system, and IBU-AG (all equivalent to 50 mg/kg IBU) were administered intragastrically with normal saline. The administration lasted for 4 weeks, during which the rats were fed a common feed and had free access to food and water. Before GFR detection, rats were anesthetized with isoflurane and a miniature imaging device composed of two light-emitting diodes, a photodiode, and a battery (MediBeacon, Mannheim, Germany) was mounted on the shaved rib cage position of rats by using double-sided tape. During the recording period (0–2 h), rats stayed awake and were kept in a cage. A single intravenous bolus of FITC-sinistrin was given 5 mg/100 g intravenously according to the body weight. Before effective measurement, the background signal was recorded for 5 min and then recorded for 2 h. The imaging device was then removed, and the fluorescence data recorded were converted into digital signals. MPD Studio software (MediBeacon, Mannheim, Germany) analyzed the data. The GFR of rats was detected by the above-mentioned *in vitro* fluorescence method on the day before gastric administration (before intervention) and on the 14th, 21st, and 28th day after gastric administration.

### 2.15. Western blot analysis

The Nrf2 and HO-1 signal molecules in rat kidney tissue were detected by Western blot analysis. The kidney tissue was homogenized and centrifuged (10,000×*g*, 10 min, 4 °C). The protein concentration was determined by a BCA protein detection kit (Beyotime, Shanghai, China), and 10% SDS polyacrylamide gel electrophoresis (SDS-PAGE) was used to separate proteins with equal quantity. Separated proteins were transferred to a polyvinylidene fluoride membrane blocked with 5% skim milk for 2 h at room temperature. The membrane was incubated overnight at 4 °C, then washed with TBS buffer containing 0.1% Tween 20, followed by incubation with horseradish peroxidase (HRP) labeled secondary antibody (1:1000) at room temperature for 2 h. The actin antibody (Beyotime, Shanghai, China) was used to ensure equal loading of protein. The quantitative blot was performed by ImageJ software (NIH, Bethesda, MD).

### 2.16. Statistical analysis

The parameters in the bioavailability experiment were calculated by DAS using the linear trapezoidal method. IBM SPSS Statistics 26 (Armonk, NY) was used for the statistical analyses. A two-tailed Student's *t*-test was performed after the one-way analysis of variance. It is statistically significant when *p*< .05 and highly statistically significant when *p*<.001.

## 3. Results and discussion

### 3.1. Screening of IBU-PSP preparation process

Binary and ternary solid dispersions were prepared with PSP as the main carrier. The effects of additive dosage, type, ball milling parameters on the solubility of IBU in an IBU delivery system were investigated, and a better solid dispersion formula and mechanochemical preparation process were obtained. The experimental conclusions are shown in Table 1.

**Table 1.** Solubility of IBU dispersions.

Entry	Sample	Ratio	Mill speed	Mill time	Solubility (mg/L)	Increase
1	IBU	–	300	4	52.22	–
2	IBU-AG	1:10	300	4	92.58	1.77
3	IBU/PSP	1:10	300	4	429.39	8.22
4	IBU/PSP/Na_2_GA	1:5:5	300	4	382.25	7.32
5	IBU/PSP/Man	1:5:5	300	4	178.60	3.42
6	IBU/PSP/GA	1:5:5	300	4	265.28	5.08
7	IBU/PSP	1:1	300	4	110.04	2.11
8	IBU/PSP	1:2	300	4	184.87	3.54
9	IBU/PSP	1:5	300	4	279.47	5.35
10	IBU/PSP	1:15	300	4	368.71	7.06
11	IBU/PSP	1:10	50	4	82.5	1.58
12	IBU/PSP	1:10	100	4	185.74	3.56
13	IBU/PSP	1:10	200	4	224.43	4.30
14	IBU/PSP	1:10	250	4	283.54	5.43
15	IBU/PSP	1:10	400	4		
16	IBU/PSP	1:10	300	0.5	100.5	1.92
17	IBU/PSP	1:10	300	1	271.23	5.19
18	IBU/PSP	1:10	300	2	323.82	6.20
19	IBU/PSP	1:10	300	4	382.25	7.32
20	IBU/PSP	1:10	300	8	349.12	6.69
21	IBU/PSP	1:10	300	12	345.88	6.62

As shown in Table 1, even though the granularity was reduced by mechanochemical grinding under the same conditions, the solubility of IBU in an aqueous solution was still very poor (only 52.22 mg/L), which was significantly lower than that of the prepared solid dispersion. The highest solubility (429.39 mg/L) of IBU in the solid dispersion was achieved by directly using PSP as a carrier, 8.22-fold higher than the API after ball milling 3.04-fold higher than the physical mixture of PSP and IBU (entries 1–6). The drug–adjuvant ratio enhanced the IBU-PSP drug delivery system solubility (entries 7–10), probably due to the expansion of the contact area between IBU and hydrophilic PSP and enhancement in the wettability of the system, thereby improving the solubility of the system. When the drug–adjuvant ratio was greater than 1:10, the solubility of the IBU-PSP drug delivery system decreased slightly, which may be related to the agglomeration of auxiliary materials under the action of mechanical force. The solubility of the IBU-PSP drug delivery system increased with the ball milling speed (entries 11–15), but when the speed of the ball milling exceeded 400 rpm, the solubility showed the trend of increasing slowly. As a result, 300 rmp was selected as the optimum ball milling frequency for saving energy. The solubility of the prepared IBU-PSP increased gradually with a prolonged milling time. When the milling time exceeded 4 h, the caking phenomenon of the system increased, and the solubility decreased. Therefore, the optimal ball milling time was set to 4 h. To sum up, a better IBU-PSP delivery system was as follows: ball milling speed of 300 rpm; ball milling time of 4 h. The solubilization multiples of the IBU-PSP drug delivery system prepared under this process could reach 8.22-fold.

### 3.2. Dissolution of IBU-PSP drug delivery system

The *in vitro* dissolution rates in simulated gastric juice and intestinal juice after giving IBU with ball milling, IBU-PSP, and IBU-AG under pH = 1.2 and pH = 6.8 were determined.

**Figure 1.**
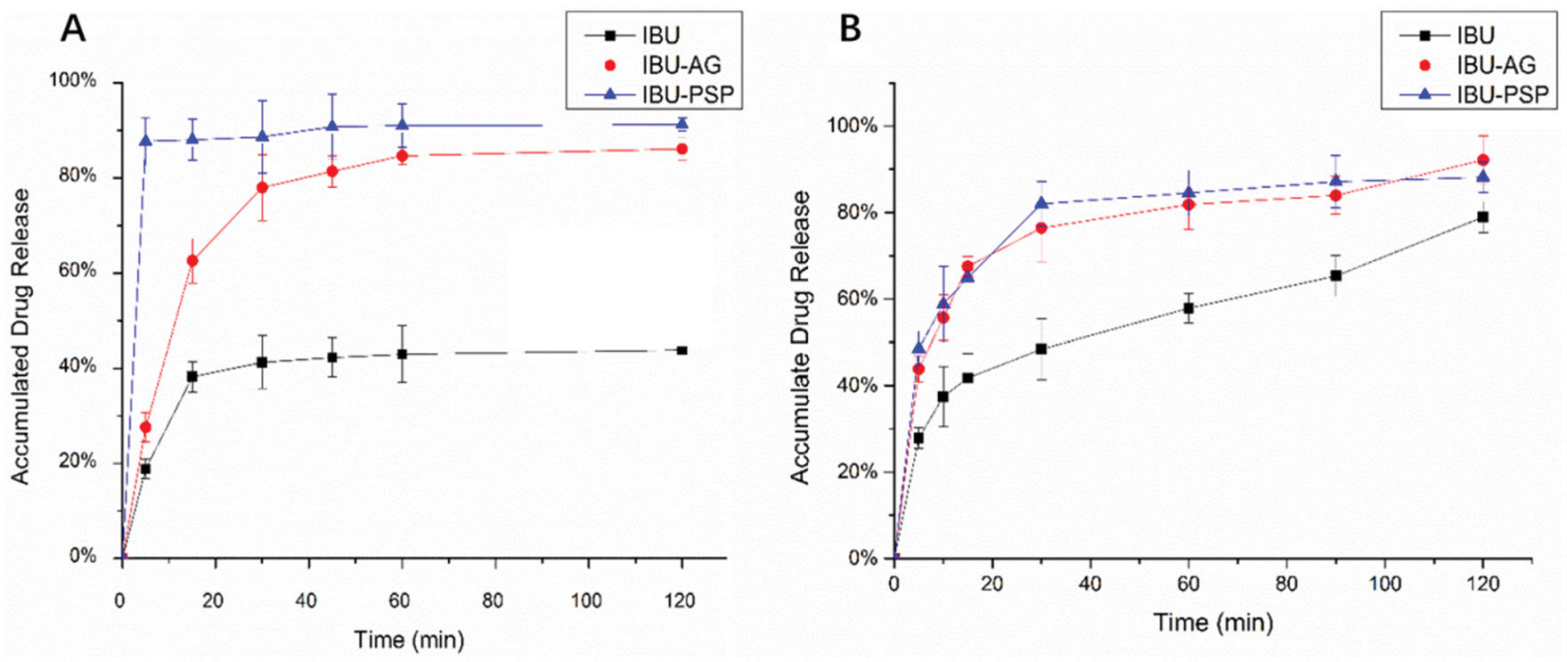
Dissolution rate at pH 1.2 (A) and pH 6.8 (B).

As shown in Figure 1, in artificial gastric juice at pH = 1.2, the maximum cumulative dissolution amount of IBU API after ball milling was only 40%, while the cumulative dissolution amount of IBU-PSP could reach nearly 85% within 5 min, and the cumulative dissolution amount of IBU-AG could also reach 80% within 40 min. In the artificial intestinal fluid with pH = 6.8, the maximum cumulative dissolution amount of IBU API after ball milling was 75%, while the cumulative dissolution amount of IBU-PSP and IBU-AG could reach 80% within 45 min. Together, these results demonstrated that the dissolution rate and cumulative dissolution amount of the IBU-PSP drug delivery system were higher than those of IBU API, which was similar to the dissolution behavior of IBU-AG in the existing rapid-release drug delivery system.

### 3.3. Characterization of physical properties of IBU-PSP solid dispersion

**Figure 2.**
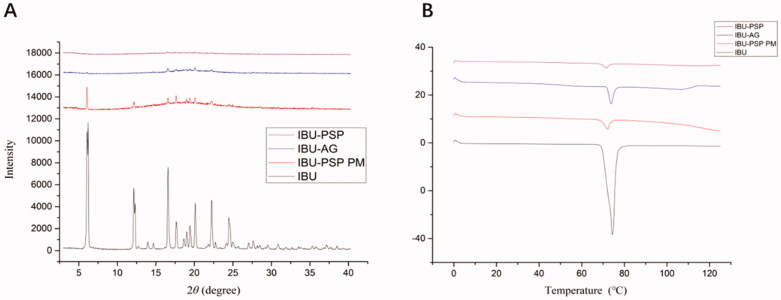
X-ray diffraction (A) and DSC (B) patterns.

According to literature reports and practical research experience, the properties of IBU-PSP were characterized by XRD and DSC, respectively. The experimental results are shown in Figure 2. DSC experiments indicated that (Figure 2(a)) the temperature at which IBU started to melt was 75.4 °C, and there was an endothermic peak at 77.09 °C. In addition, the melting temperature of IBU decreased slightly with greatly decreased absorption strength in the DSC spectrum of IBU-PSP. The XRD conclusion is reported in Figure 2(b). Many characteristic peaks were observed in the IBU crystal at 2*θ* angle within 30°, and the XRD characteristic peaks of IBU-PSP and IBU-AG almost disappeared. The above results suggested that IBU in IBU-PSP was in an amorphous state, and therefore the water solubility was improved.

### 3.4. Characteristics of IBU-PSP solid dispersion

**Figure 3.**
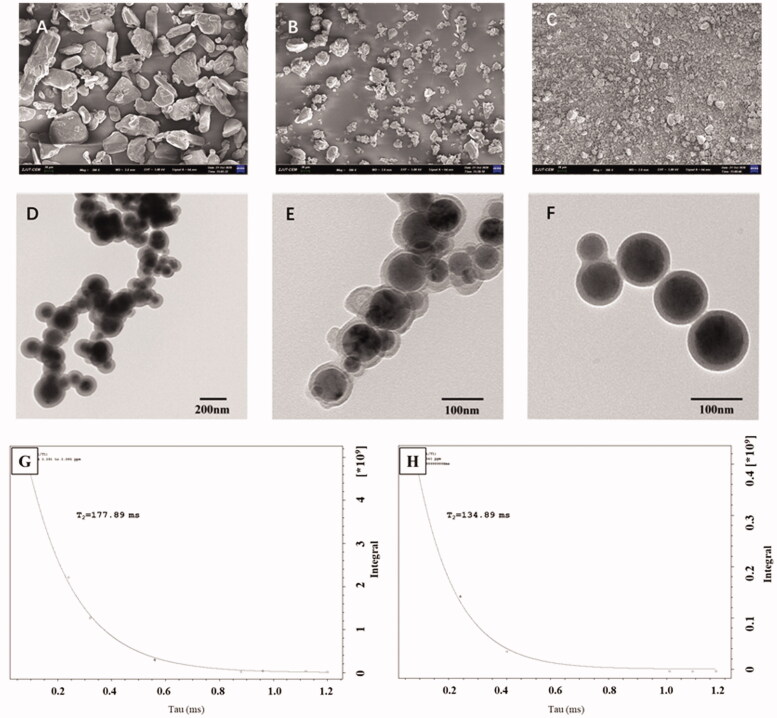
SEM photographs (A: IBU (×200); B: PSP (×200); C: IBU-PSP BM (×200)), TEM photographs (D: PSP BM (×500); E: PSP BM (×1000); F: IBU-PSP BM (×1000)) and the spin–spin *T*_2_ relaxation time of IBU-PSP physical mixture (G); IBU-PSP BM (H).

SEM and TEM were used to analyze the solid dispersion of IBU-PSP and its microscopic state in an aqueous solution. As shown in Figure 3, SEM images (4-1) of IBU, Polygonatum polysaccharide, IBU-PSP, and physical mixture showed that the IBU and PSP after ball-milling had a different morphology and a larger particle size. After being prepared into IBU-PSP by mechanochemistry, the crystal structure of IBU was destroyed and fused with Polygonatum polysaccharide particles to form a uniform solid dispersion consisting of uniform small particles and aggregates. TEM images of IBU-PSP in an aqueous solution further showed that the IBU-PSP delivery system formed spherical microcapsules in an aqueous solution with clear boundaries and uniform and regular particles indicating that IBU-PSP can form spherical microcapsules in an aqueous solution. Furthermore, nano-particle size analysis showed that the average particle size of nano-particles of IBU-PSP formed in aqueous solution was 109.1 nm, which was close to 100 nm, indicating that the IBU-PSP delivery system may be self-assembled as spherical nano-particles in solution. The *ζ* potential was measured as −33.1 ± 0.7 mV, −32.6 ± 0.9 mV after being stored in the dark at room temperature for one week. The absolute value of *ζ* potential was greater than 30 mV, suggesting that the nano-particles had good stability in an aqueous solution.

As mentioned in the experimental section of 2.10, the spin–spin *T*_2_ relaxation time of the small molecule protons is very sensitive to the intermolecular interactions and the diffusivity of the molecules (Emsley et al., 1966; Popova et al., 2004). We measured the relaxation times *T*_2_ of IBU proton (3.377 ± 0.05 ppm) of the physical mixture of IBU and PSP and IBU-PSP made by a mechanochemical method in the aqueous solution. The *T*_2_ relaxation time (see Figure 3(G,H)) of the complexes was decreased compared to IBU in the physical mixture, reflecting the formation of the complexes. It should be noted that all kinetics in the relaxation experiments showed mono-exponential time-dependence, which points to a rapid exchange of the ‘guest’ molecules between the complex and solution. Due to complex formation, the relaxation time of IBU in IBU-PSP (134.89 ms) was lower than that of the IBU in the physical mixture (177.9 ms), suggesting that in contrast to lipophilic molecules which penetrate the core of the PSP globule, IBU molecules are located outside the complex or near the outer surface of the PSP globule which had been recognized as an intermolecular complex.

### 3.5. Pharmacokinetic characteristics of IBU-PSP

**Figure 4.**
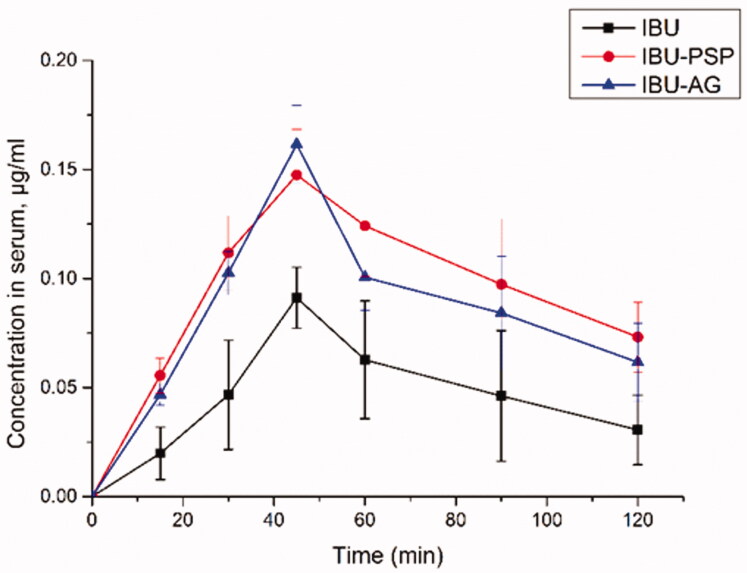
Serum concentration of IBU (*n* = 6).

With IBU-PSP, IBU-Polygonatum polysaccharide physical mixture, IBU-AG, and IBU API as research objects and rats as the experimental animal model, the rat plasma concentration of samples at different periods was evaluated using the technique described in Sections 2.1–2.11. The experimental results are shown in Figure 4. Based on the plasma concentration of IBU and IBU solid dispersion after oral administration, the *T*_max_ of all experimental groups was the same, which was 45 min. However, compared with the IBU group, the *C*_max_ of oral IBU-PSP was about 1.5-fold higher than API, close to the reported oral plasma concentration of IBU-AG solid dispersion with high bioavailability. Furthermore, the changes in plasma concentration in each group were analyzed by DAS software, and the oral bioavailability parameters of API, IBU-AG, and IBU-PSP were obtained. Moreover, the significance of each parameter was analyzed by PLASM software.

As shown in Table 2, after oral administration of IBU-PSP and IBU-PSP, the maximum plasma concentration of IBU was significantly higher than that of the IBU API group. Moreover, the AUC of IBU in the IBU-PSP group was about 2.52-fold higher than that in the IBU group and about 30% higher than that in the IBU-AG group, indicating that IBU-PSP could significantly improve the bioavailability of IBU *in vivo* and its effect was better than the reported high-bioavailability IBU-AG solid dispersion. Further studies showed that *t*_1/2_ of the IBU-PSP delivery system group was significantly longer than that of the IBU and the IBU-AG groups, which indicated that IBU-PSP could appropriately prolong the IBU short-term efficacy. Thus, the bioavailability data showed that IBU-PSP could improve the oral bioavailability of IBU and slightly prolong the efficacy time.

**Table 2.** Pharmacokinetics parameters after oral introduction of IBU (*n* = 6).

Parameters	IBU	IBU-PSP	IBU-AG
*t*_1/2_, min	47.41 ± 3.18	74.26 ± 4.57	53.38 ± 3.98
*T*_max_, min	45.00 ± 0.00	45.00 ± 0.00	45.00 ± 0.00
*C*_max_, mg/L	0.0912 ± 0.0046	0.1475 ± 0.0081	0.1615 ± 0.0078
AUC_0–∞_ (mg·min)/L	7.61 ± 0.80	19.19 ± 1.74	14.77 ± 1.19
AUMC_0–∞_ (mg·min^2^)/L	744.37 ± 78.35	2503.68 ± 294.47	1571.12 ± 197.20

### 3.6. Anti-inflammatory pharmacodynamic experiment of IBU-PSP

**Figure 5.**
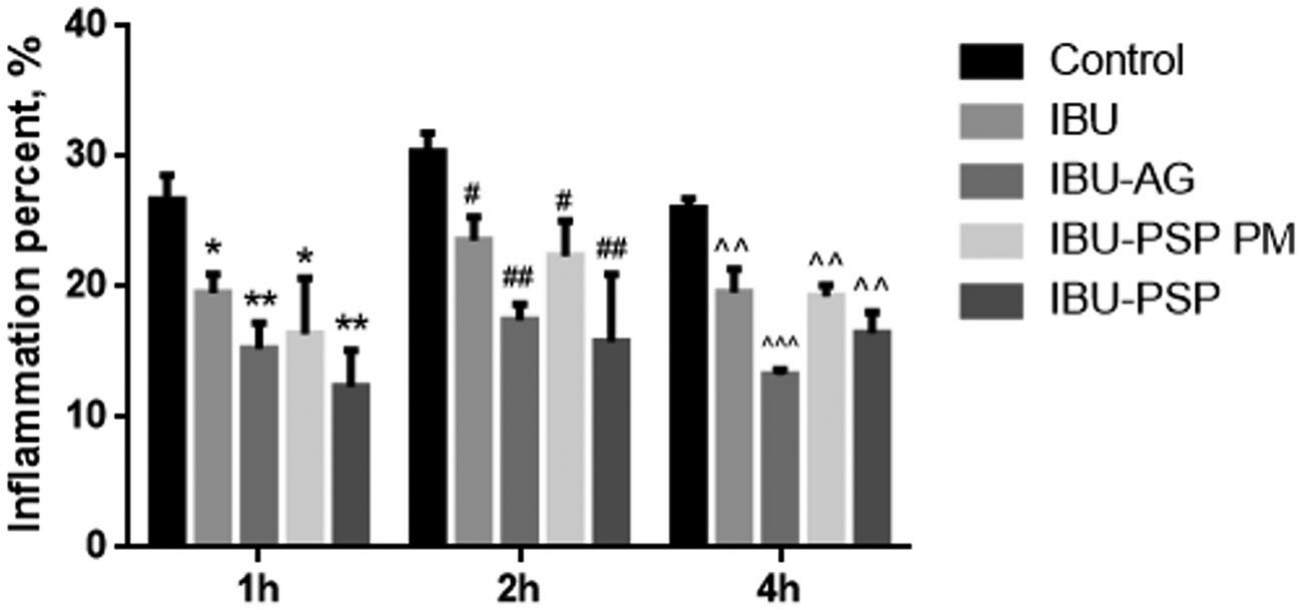
Swelling degree of rat toes (*n* = 10, *p, ^#^*p*<.05 vs. control, **p, ^##^*p*, ^^*p*<.01 vs. control, ^^^*p*<.01 vs. control at different time).

The combination of anti-inflammatory animal experiments indicated that IBU-PSP could boost the anti-inflammatory impact of IBU. A rat carrageenan-induced edema model was used to assess the anti-inflammatory effects of IBU, IBU-AG, IBU-PSP, and a physical combination of IBU and Polygonatum polysaccharide at a ratio of 1:10. As shown in Figure 5, using the same administration dose, compared with API and the physical mixture, the degree of foot swelling of rats in the IBU-PSP and IBU-AG groups was significantly reduced (*p*< .01, *p*< .001), and IBU-PSP was better than IBU-AG in reducing foot swelling. According to bioavailability and preliminary anti-inflammatory tests, it could be concluded that IBU-PSP could significantly increase the anti-inflammatory effect of IBU, and the effect was closely related to the increase in plasma concentration and bioavailability of IBU. However, IBU is metabolized by the kidney after it is removed, and the increase in plasma concentration would inevitably lead to increased renal toxicity. The impact of IBU-PSP in reducing IBU-induced kidney damage will be investigated further in the follow-up study using acute and chronic renal toxicity.

### 3.7. IBU-PSP reduces acute nephrotoxicity induced by IBU

**Figure 6.**
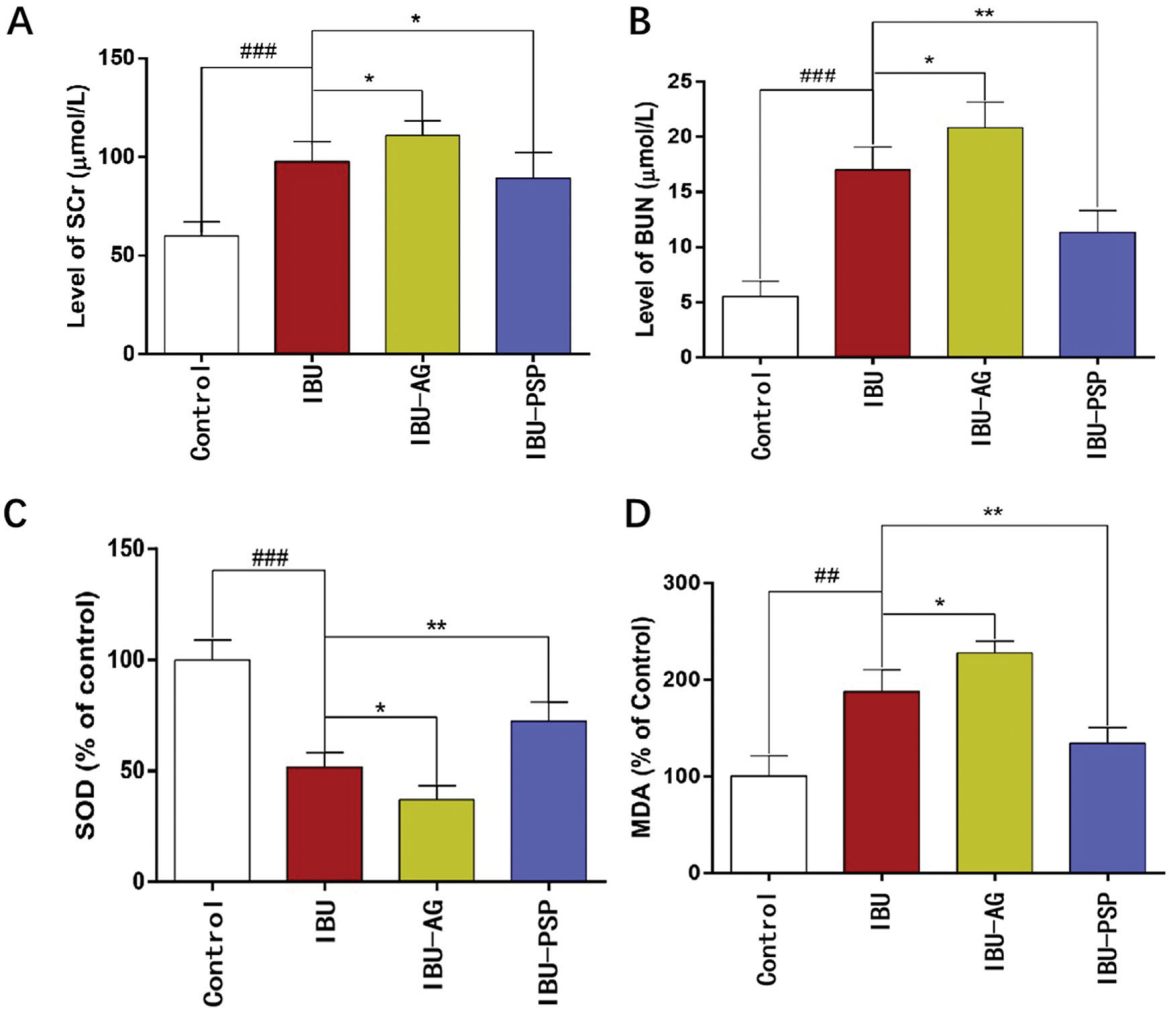
Serum creatinine (A), urea nitrogen (B) in blood and SOD activity (C), MDA concentration (D) in kidney of rats (*n* = 10; *p<.1 vs. IBU, **p<.01 vs. IBU, ^##^*p*<.01 vs. control, ^###^*p*<.001 vs. control).

The effects of IBU-PSP, IBU-AG, and IBU on reducing acute renal toxicity caused by IBU were evaluated by increasing the dosage of IBU and IBU preparations. After oral administration with the maximum dosage of IBU to cause renal damage, renal damage in the IBU group, IBU-AG, and IBU-PSP group was investigated by blood biochemistry and renal tissue-related parameters. The experimental results are shown in Figure 6. After administration, the contents of SCr (*p*< .001) and urea nitrogen increased significantly (*p*< .001), and the MDA content in kidney tissue increased significantly (*p*< .01), which was about twice as high compared to that in the normal group. However, SOD activity in kidney tissue in the IBU group decreased significantly, which was only 1/2 of that in the normal group (*p*< .001), thereby indicating that oral administration of IBU at this dose could induce oxidative stress-related injury in kidney tissue of rats. In terms of the rats in the IBU-AG administration group, it was observed that high-dose medication could cause depression and the decrease in the vitality of rats, and the contents of SCr (*p*< .05) and urea nitrogen (*p*< .05) were higher than those of the IBU group.

Furthermore, the SOD activity (*p*< .05) in kidney tissue was significantly lower than that in the IBU group, while the MDA content was higher than that in the IBU group. These findings indicated that after IBU-AG administration, the bioavailability of IBU was improved, and at the same time, kidney injury caused by IBU was increased, thereby causing more serious oxidative stress in the kidney. In rats in the IBU-PSP administration group, it was observed that high-dose administration did not reduce the activity of rats. The contents of SCr, urea nitrogen, and MDA in the kidney were significantly lower than those in the IBU and IBU-AG groups. In contrast, the SOD enzyme activity in kidney tissue of rats was significantly higher than that of IBU (*p*< .01). Thus, serum and kidney parameters indicated that IBU-PSP could alleviate acute nephrotoxicity caused by IBU.

**Figure 7.**
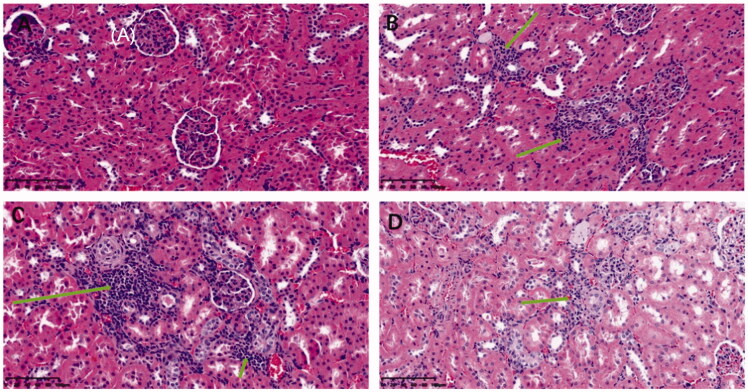
HE Chromatic graph of glomerular mesangial cells (A: normal group, B: IBU group, C: IBU-AG group, D: IBU-PSP group).

In addition, after experiments, the glomeruli of rats in each group were stained with HE to study changes in the kidney microstructure in each group and evaluate this from the perspective of kidney morphology. The pathological damage of kidney tissue was analyzed through observing the glomerular volume and mesangial matrix, and the pathological staining results are shown in Figure 7. It was demonstrated that the glomerular volume of normal rats was normal and that the structure of capillary loops was clear. No pathological changes, such as mesangial hyperplasia were found. Compared with the normal group, the glomerular volume of the IBU and IBU-AG groups increased significantly, increasing cell number. Mesangial cells proliferated, and the mesangial matrix increased with the thickening of the basement membrane. Tubular epithelial cells presented vacuolar degeneration, and the changes in glomerular morphology of the IBU-AG group were more obvious. However, pathological sections of kidney tissue in the IBU-PSP administration group showed that kidney inflammation caused by IBU was improved, which was close to the glomeruli of healthy mice. Altogether, the above-mentioned experimental conclusions indicated that IBU-PSP could inhibit acute renal damage caused by a large dose of IBU.

### 3.8. Evaluation of chronic nephrotoxicity

Subsequently, the inhibitory effect of IBU-PSP on chronic nephrotoxicity induced by IBU was studied. IBU, IBU-AG, and IBU-PSP were given orally according to the normal dosage for 28 days, and the GFR of rats was evaluated by an *in vivo* fluorescence tracing method. The chronic nephrotoxicity of the IBU-PSP delivery system was studied in the blank control group, IBU, and IBU-AG delivery system as a control. The main features of long-term chronic kidney damage included changes in the GFR. Therefore, we studied the kidney damage caused by long-term administration of IBU in IBU-PSP via investigating GFR in rats.

**Figure 8.**
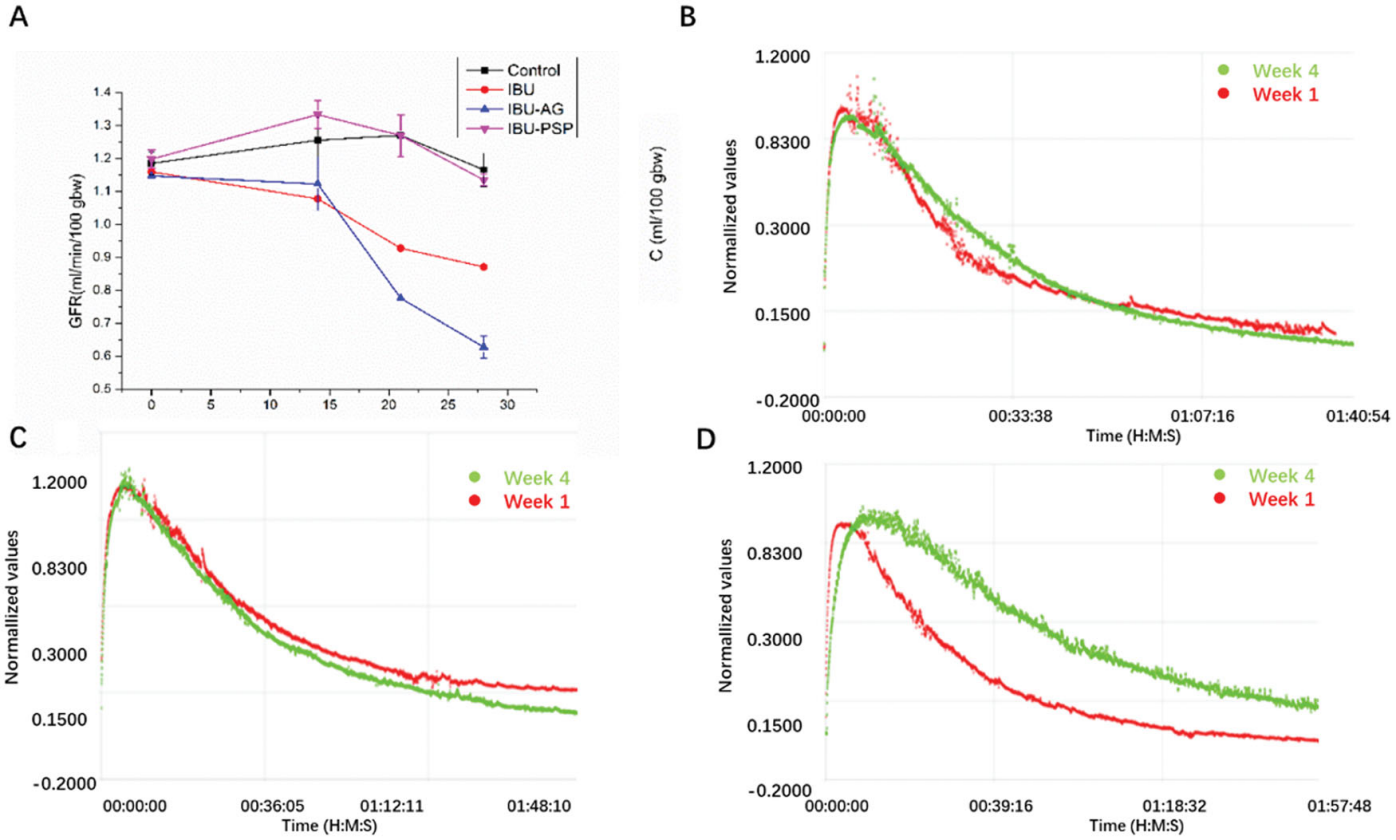
(A) Change curve of GFR within 28 days; (B) GFR comparison curve at the beginning and end of control group; (C) GFR comparison curve at the beginning and end of IBU-PSP group; (D) GFR comparison curve at the beginning and end of IBU-AG group.

As shown in Figure 8, no significant differences in the GFR value of rats were observed in each group before administration. After 14 days of continuous administration, the GFR of the IBU and IBU-AG groups decreased slightly compared with that of the blank control group. However, the GFR of the IBU-PSP administration group was better than that of the blank group, which indicated that PSP played a protective role in the kidney. After 28 days of continuous administration, compared with the normal group, the GFR in the IBU group and IBU-AG group decreased continuously, and a more significant decrease was found in the IBU-AG group. In terms of the IBU-PSP group, the GFR changes were close to those in the normal group, which suggested that long-term IBU administration would lead to chronic and long-term renal damage in rats. For the IBU-AG group, the slower decay of the clearance curve in week 4 compared to week 1 means reduced GFR after 28 days' treatment, indicating that chronic renal damage was aggravated after improving the bioavailability. However, in the IBU-PSP group, renal damage was greatly reduced, and long-term medication for 28 days did not cause a significant reduction in renal function.

### 3.9. Mechanism of IBU-PSP in reducing renal toxicity induced by IBU

It has been reported that NSAIDs, such as IBU, can inhibit cyclooxygenase-2 (COX-2) in the kidney, and COX in the kidney is closely related to the excretion of salt and water and the regulation of renal blood flow, which leads to a decrease in the GFR. At the same time, NSAIDs, such as IBU, can cause NADPH oxidase expression, renal cell apoptosis, up-regulation of pro-inflammatory cytokines in the kidney (Basivireddy et al., 2004; Fattori et al., 2017), resulting in oxidative stress and damage to the normal function of kidney cells. The action mentioned above sites is closely related to IBU-induced nephrotoxicity. Therefore, we preliminarily explored the renal protective mechanism of IBU-PSP according to the protein expression of Nrf2/Ho-1, NF-KB/TNF-α, and COX-2 in rat kidneys after administration of IBU-PSP.

**Figure 9.**
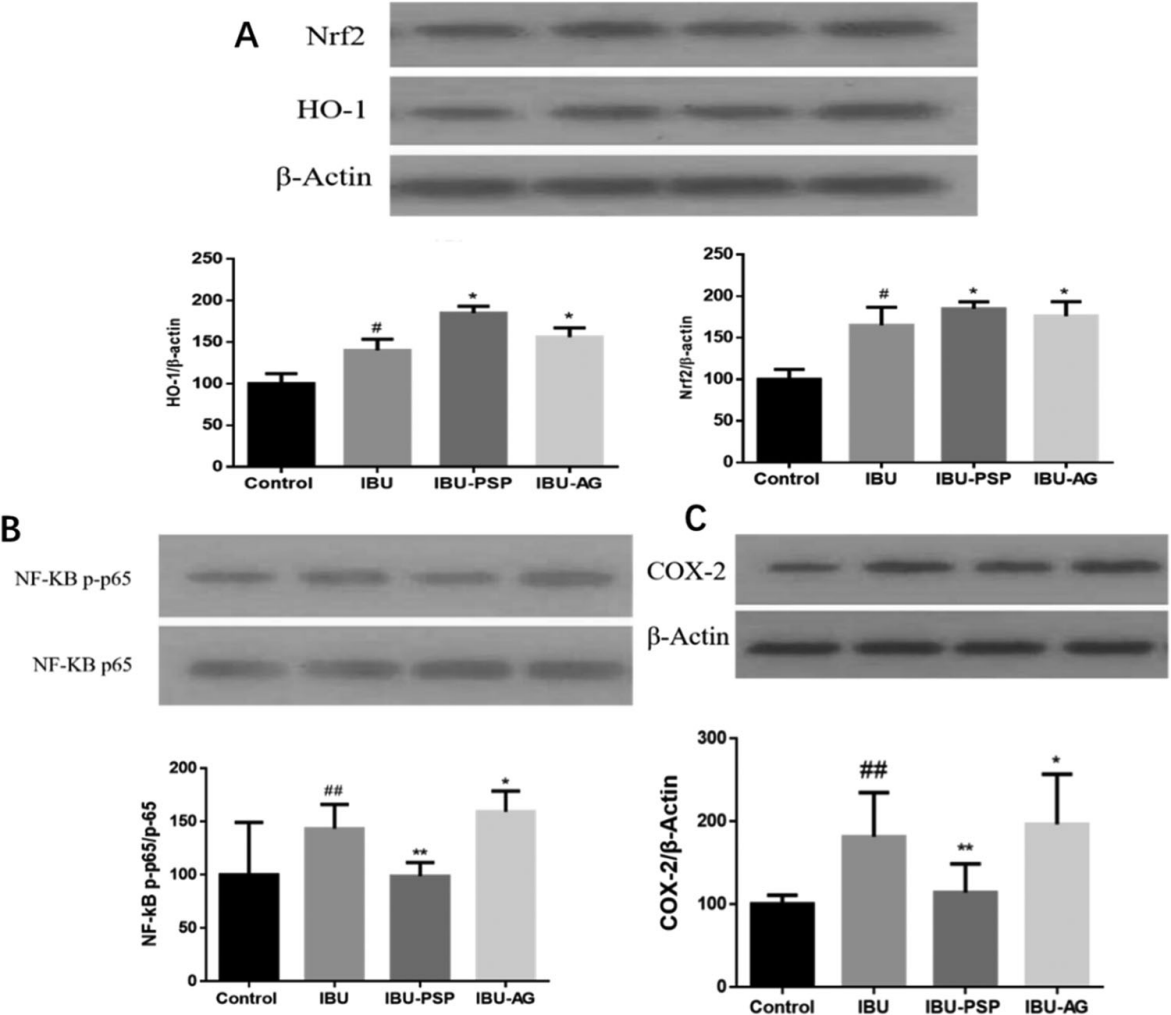
Expression of Nrf2/HO-1 protein (A), NF-KB/TNF-α and COX-2 in rat kidney (*n* = 10; ^#^*p*<.05 vs. control, *p<.1 vs. control, ^#^^#^*p*<.01 vs. control, **p<.01 vs. control).

As shown in Figure 9(A), the Nrf2 content in kidney tissues of the IBU group, IBU-AG group, and IBU-PSP group increased significantly (*p*< .05). Combined with the detection of oxidative stress levels in kidney tissues of rats, the increase in Nrf2/Ho-1 content may be related to the activation of oxidative stress of the kidney by IBU. For the IBU-PSP group, the content of MDA in the kidney was not increased when the expression of Nrf2/Ho-1 was up-regulated. The results indicated that PSP could quench reactive oxygen species (ROS) produced by oxidative stress, thus alleviating oxidative damage of the kidney caused by IBU. As shown in Figure 9(B), compared with the normal group, the contents of NF-KB and TNF-α in kidney tissue of the IBU group and IBU-AG group increased significantly. In particular, the expression of NF-KB and TNF-α in kidney tissue of the IBU-AG group was significantly higher than those of the IBU group, suggesting that improving the bioavailability of IBU could aggravate the inflammatory level of the kidney. However, the contents of NF-KB and TNF-α in kidney tissue of the IBU-PSP group were significantly lower than those of the IBU and IBU-AG groups, which were closely related to inflammatory immune responses in kidney tissue. The experimental results demonstrated that IBU-PSP could reduce the level of kidney inflammation caused by IBU. As shown in Figure 9(C), compared with the normal group, the expression of COX-2 in kidney tissues of the IBU group and IBU-AG group was up-regulated, indicating that IBU inhibited COX-2 activity and increased the verification of kidney tissues, while the expression of COX-2 in kidney tissues of IBU-PSP group was close to that of the normal group, with a slight increase, indicating that PSP in IBU-PSP reduced the inhibition of IBU on COA-2 in the kidney, thereby reducing the level of inflammation. It was suggested that the IBU-PSP drug delivery system could protect the kidney.

According to the research on the three key proteins of kidney damage caused by NSAIDs, IBU was similar to other NSAIDs, and its nephrotoxicity was closely related to the inhibition of COX-2 activity, inflammation, and oxidative stress. In the IBU-AG group, solid dispersion improved the bioavailability of IBU, and the concentration of IBU in the kidney increased significantly, with increased expression of COX-2 and intensified inflammatory factors and oxidative stress. At the same dosage, the nephrotoxicity generated by IBU-AG was higher than IBU's. However, in the IBU-PSP group, the carrier of PSP presented excellent renal protection function, which represented as the inhibition on the up-regulation of COX-2 expression, quenching the active oxygen produced by oxidative stress and the inhibition on the production of inflammatory factors, thus improving the bioavailability of IBU and reducing the renal damage caused by IBU.

## 4. Conclusions

This study directly prepared IBU and Polygonatum polysaccharides into solid dispersion IBU-PSP via mechanochemical technology. Through optimizing the mechanochemical technology, the solubility of IBU in an aqueous solution increased 8.22-fold compared with that of IBU API, which was close to that of IBU in IBU-AG. Dissolution test results further showed that the dissolution and cumulative dissolution of IBU-PSP were better than those of IBU, API, and IBU-AG. Furthermore, the morphology of IBU-PSP solid dispersion was characterized by DSC, XRD, DLS, SEM, and TEM. The experimental results showed that IBU and Polygonatum polysaccharide formed amorphous solid dispersion under mechanochemical action, and stable intermolecular complex could be formed in solution, with an average particle size of 109.1 nm and *ζ* potential of −32.6 ± 0.9 mV.

Pharmacokinetics and anti-inflammatory experiments indicated that IBU-PSP could improve the plasma concentration and bioavailability of IBU, which was 1.2-fold higher than that of IBU-AG. A carrageenan-induced rat foot swelling model evaluated the anti-inflammatory effects of IBU-PSP, physical mixture, and IBU-AG. It was found that the IBU-PSP drug delivery system could enhance the anti-inflammatory effect. Nephrotoxicity experiments further indicated that the intake of a large dose of IBU in a short time would produce acute nephrotoxicity, which was manifested by the increase in SCr and urea nitrogen, and a decrease in GFR, while IBU-AG could lead to the increase in plasma concentration of IBU, which directly increased the toxicity of IBU to the kidney. Although IBU-PSP improved the plasma concentration and bioavailability of IBU, the carrier of PSP could protect the kidney, thereby greatly reducing acute and chronic kidney damage caused by IBU. At last, through studying the mechanism of IBU-PSP inhibiting kidney damage, it was found that PSP, as a drug carrier, could activate the expression of Nrf2/HO-1 signal molecules in the kidney, regulate the activity of downstream antioxidant enzymes, and play a role in anti-oxidative stress. In addition, an anti-inflammatory effect could be achieved by reducing NF-kB/TNF- in the kidney, inhibiting the expression of COX-2 in the kidney, and achieving an effect of preventing kidney damage caused by the IBU.

## Data Availability

The data and materials generated and analyzed during the current study are available from the corresponding author on reasonable request.
